# Anatomical study of the coffee berry borer (*Hypothenemus hampei*) using micro-computed tomography

**DOI:** 10.1038/s41598-019-53537-z

**Published:** 2019-11-20

**Authors:** Ignacio Alba-Alejandre, Javier Alba-Tercedor, Fernando E. Vega

**Affiliations:** 1 Department of Zoology, Faculty of Sciences, University of Granada, Campus de Fuentenueva, 18071 Granada, Spain; 2 Sustainable Perennial Crops Laboratory, United States Department of Agriculture, Agricultural Research Service, Beltsville, MD 20705 USA

**Keywords:** Biological techniques, Entomology

## Abstract

Traditionally, the study of anatomy in insects has been based on dissection techniques. Micro-computed tomography (micro-CT) is an X-ray based technique that allows visualization of the internal anatomy of insects *in situ* and does not require dissections. We report on the use of micro-CT scans to study, in detail, the internal structures and organs of the coffee berry borer (*Hypothenemus hampei*), the most damaging insect pest of coffee worldwide. Detailed images and videos allowed us to make the first description of the aedeagus and the first report of differences between the sexes based on internal anatomy (flight musculature, midgut shape, hindgut convolutions, brain shape and size) and external morphology (lateral outline of the pronotum and number of abdominal tergites). This study is the first complete micro-CT reconstruction of the anatomy of an insect and is also the smallest insect to have been evaluated in this way. High quality rendered images, and additional supplementary videos and 3D models are suitable for use with mobile devices and are useful tools for future research and as teaching aids.

## Introduction

Ever since the introduction of coffee from Africa to various continents, it has become an important commodity not only to producing countries, but also to billions of consumers that take delight on its flavour, aroma, and stimulatory effects^1^. Amongst the many agronomic problems faced by coffee producers, insect pests and plant pathogens are of paramount importance^2^. Climate change is also posing an enormous challenge to the continuity of commercial coffee production and to the survival of wild coffee species^3,4^.

The coffee berry borer, *Hypothenemus hampei* (Ferrari) (Coleoptera: Curculionidae: Scolytinae: Cryphalini), is the most economically important insect pest of coffee due to its cryptic life cycle inside the coffee berry, which makes it quite hard to manage^5^. The insect is currently present in most coffee producing countries^5^ and worldwide losses are likely over US$500 million, with yearly losses in Brazil alone estimated at US$215–358 million^6^. The insect has also been reported to have infested vast areas, e.g., over 715,000 ha in Colombia by 1998^7^ and more than 800,000 ha by 2002^8^. Even though > 1,800 papers have been published on the coffee berry borer^9^, knowledge about its internal anatomy is restricted to a handful of papers that use dissection techniques^10–14^.

Insect dissection techniques were first used more than 400 years ago by Aldrovandi^15^ and Malpighi^16^. Even though dissections have most definitely been useful, resulting in thousands of papers on the internal anatomy of insects, they result in an inevitable distortion of the internal structures and organs. A relatively new technique based on X-rays, known as micro-computed tomography (micro-CT), allows visualization of the insect’s internal anatomy *in situ*, without the need for dissection; results have been validated by comparing them with classical destructive methodologies^17,18^.

We present a detailed study of all the anatomical structures and organs in the coffee berry borer using micro-CT, with the exception of the respiratory system, which is presented separately^19^. This is in contrast to other insect-related micro-CT papers that focus on specific aspects such as cephalic anatomy^20^, metamorphosis^21,22^, tracheal systems^23–28^, mycetangia^29–31^, mandibular muscles^32^, the alimentary canal^33^, respiratory volume^34^, genitalia^35^ and the brain^36^. Together with the study on the respiratory system^19^, this paper represents the first holistic view of the entire anatomy of the coffee berry borer, and to the best of our knowledge, is the smallest insect to have been subjected to micro-CT.

## Results

**Figure 1:**
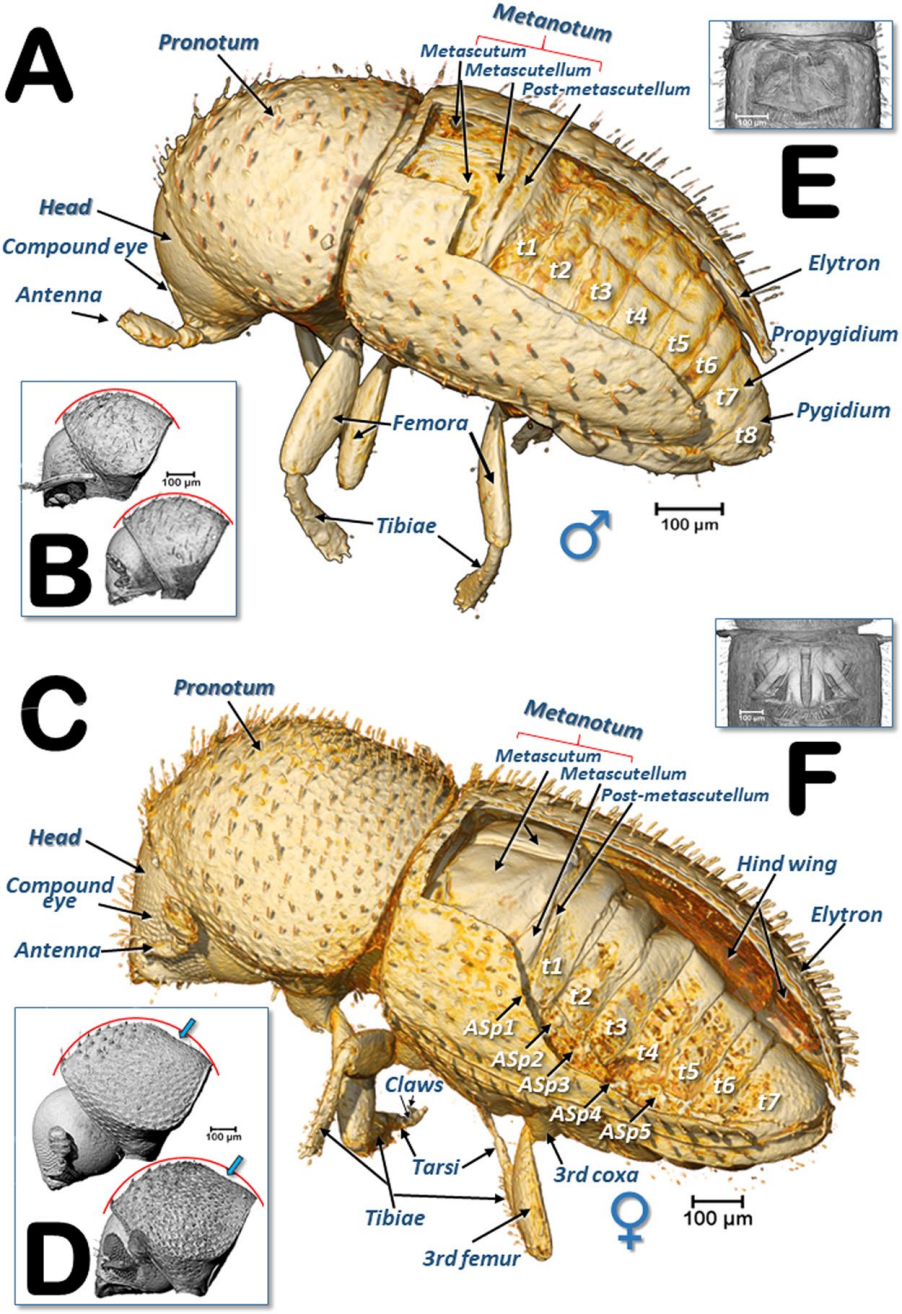
Left dorso-lateral views of a male (**A**) and a female (**C**) coffee berry borer. Elytra have been removed using software thereby revealing the dorsal part of the thoracic and abdominal segments. Abdominal tergites (t) and the position of the abdominal respiratory spiracles (ASp) are labelled sequentially (terminology after Hopkins^39^). Insets (**B**,**D**) show a uniformly curved pronotal disc in males (**B**) in contrast to a concave depression on the posterior pronotal third in females (**D**; blue arrows). Note different scales were used for each sex. The meso- and metanotum were removed by software to observe the reduced flight muscles in the male (**E**) in contrast to conspicuous flight muscles in the female (**F**).

Previously unreported external differences between the sexes were found: males had eight abdominal tergites (Fig. 1A) and, in lateral view, the outline of the pronotal disc appeared uniformly curved (Fig. 1B); the females had seven abdominal tergites (Fig. 1C) and the outline of the pronotal disc had a slight concave depression (Fig. 1D).

**Figure 2:**
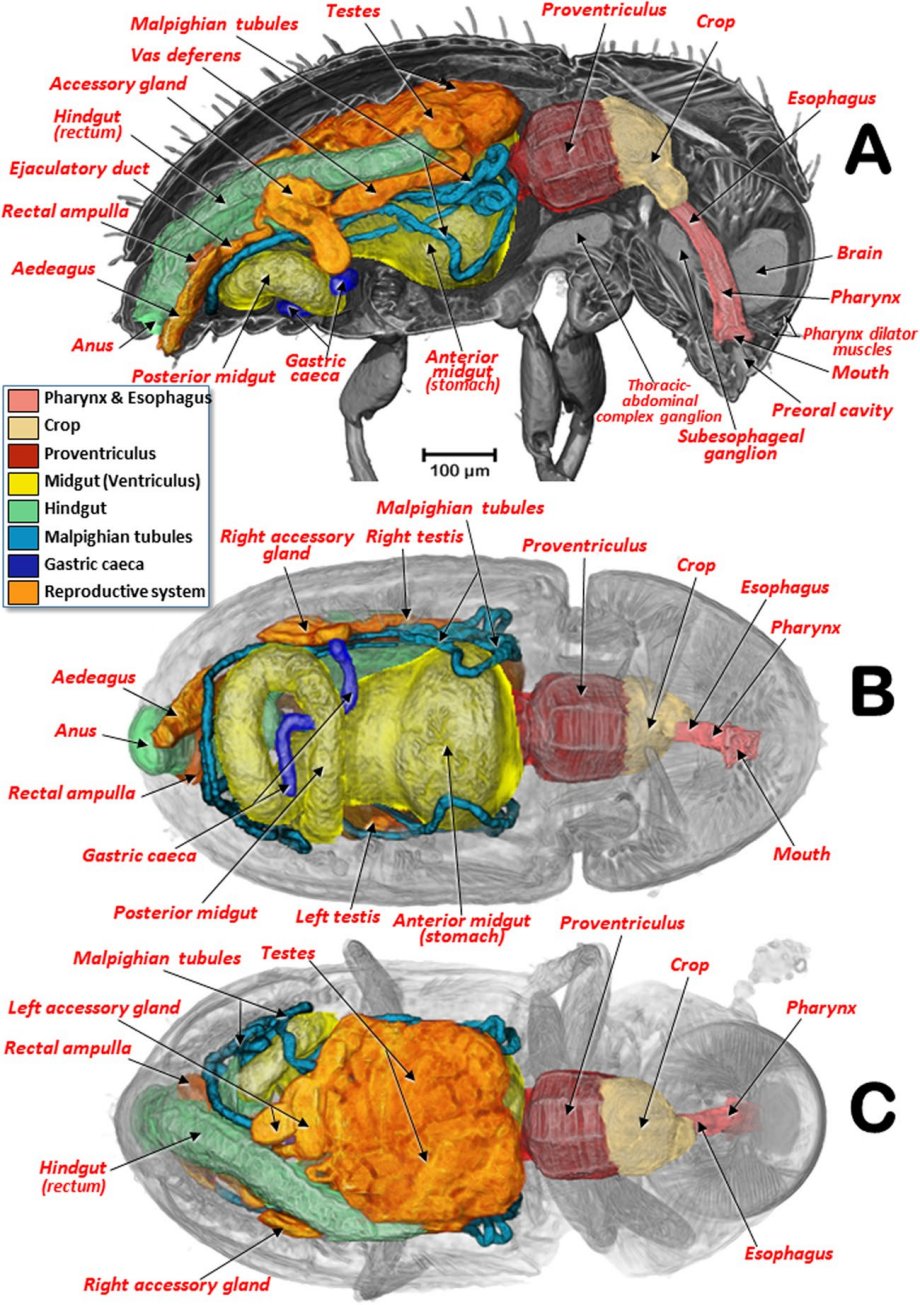
Internal anatomy of a male coffee berry borer: right-lateral (**A**), ventral (**B**), and dorsal (**C**) views. To enhance the actual anatomical position of the different organs, the body (except the internal organs) have been rendered transparent (**B**,**C**) by depleting the opacity values using Amira software.

**Figure 3:**
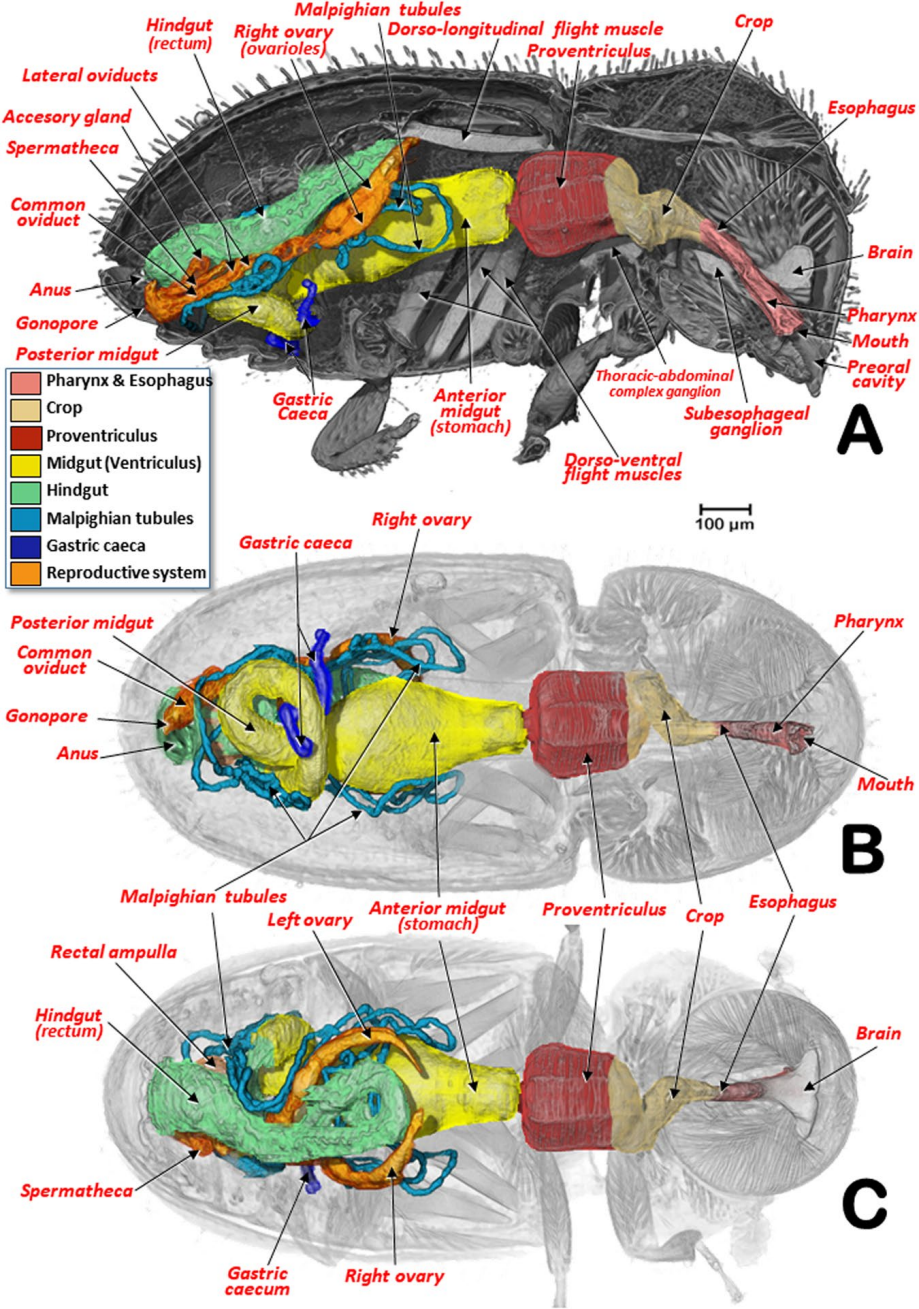
Internal anatomy of a female coffee berry borer: right-lateral (**A**), ventral (**B**), and dorsal (**C**) views. To enhance the actual anatomical position of the different organs, the body (except the internal organs) has been rendered transparent (**B**,**C**) by depleting the opacity values using Amira software.

**Figure 4:**
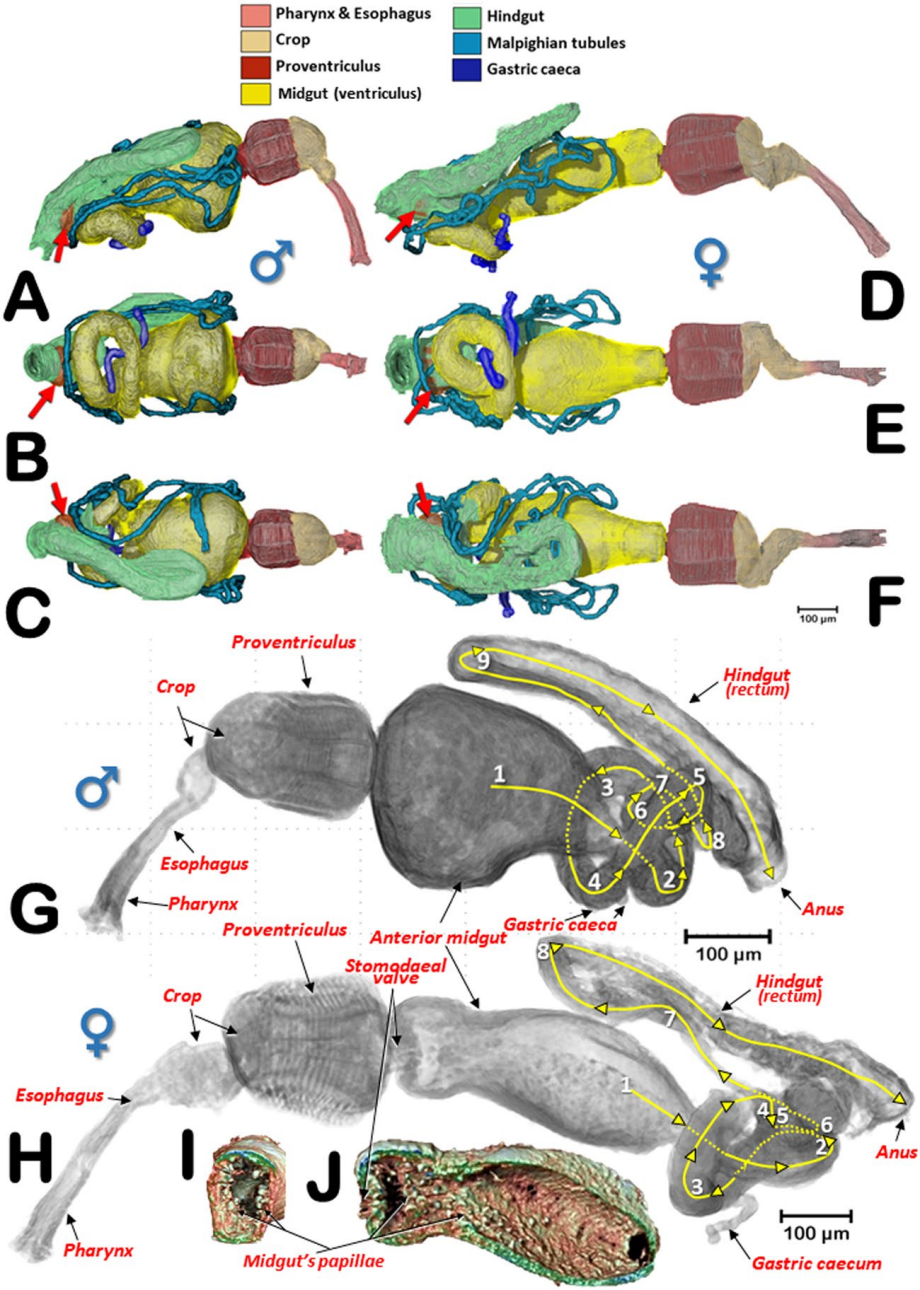
Alimentary canal of a male (**A**,**B**,**C**) and a female coffee berry borer (**D**,**E**,**F**): right-lateral (**A**,**D**), ventral (**B**,**E**), and dorsal (**C**,**F**) views. Red arrows indicate the rectal ampulla. Lateral views of the male (**G**) and female (**H**) alimentary canals with the trajectories and convolutions of the midgut and hindgut shown in yellow. Each time the digestive tracts began a new trajectory curve, the new tract is marked with an incremental number. Anterior-transversal (**I**) and sagittal (**J**) cuts close to the stomodaeal valve of the midgut reveal the internal papillae.

**Figure 5:**
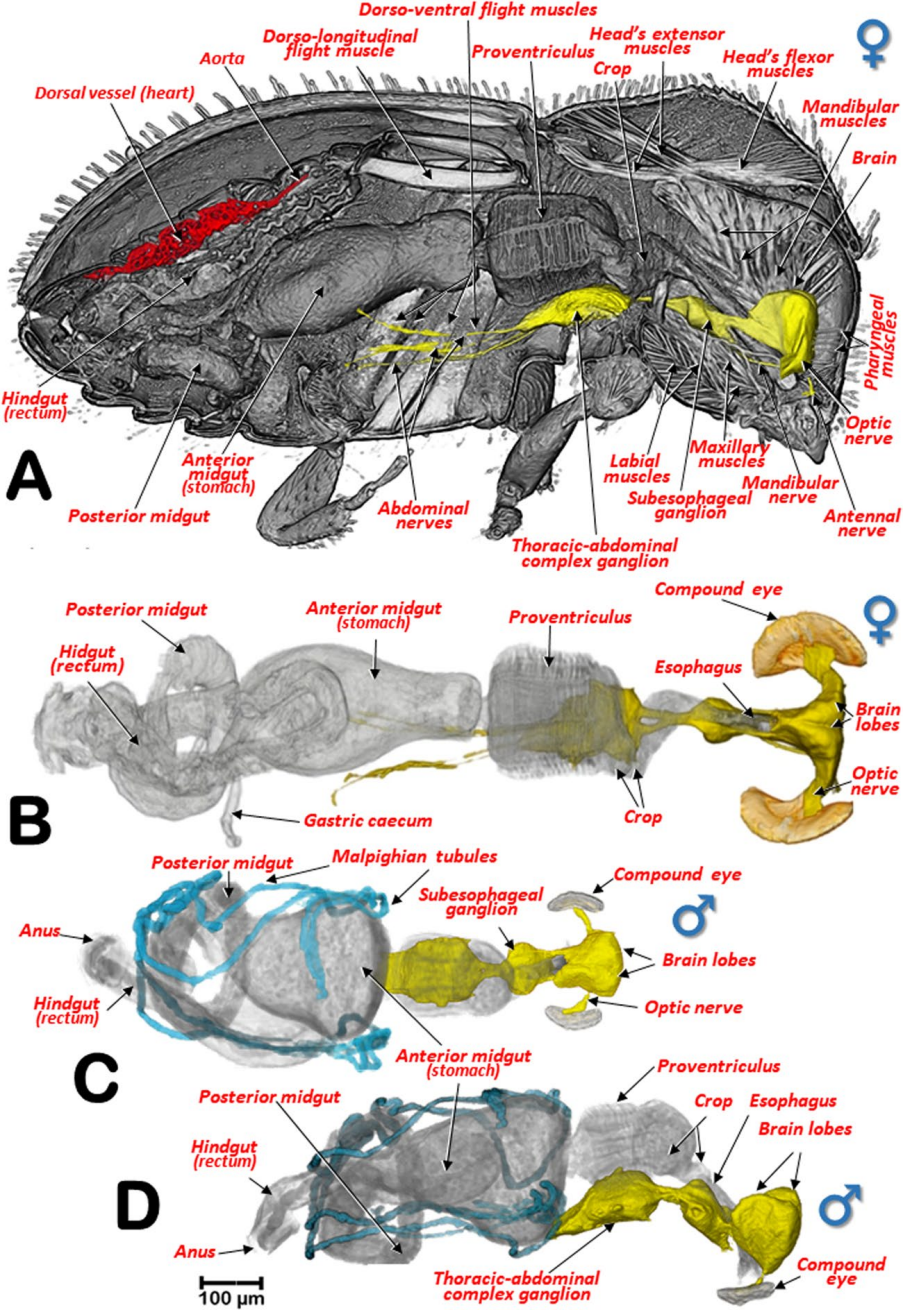
Right-sagittal view of the internal anatomy of the female, showing the main musculature, dorsal vessel and nervous system (**A**), and a dorsal view of the digestive and nervous systems (**B**). Dorsal (**C**) and right-lateral (**D**) views of a male focusing on the positioning of the nervous system in relation to the digestive system.

**Figure 6:**
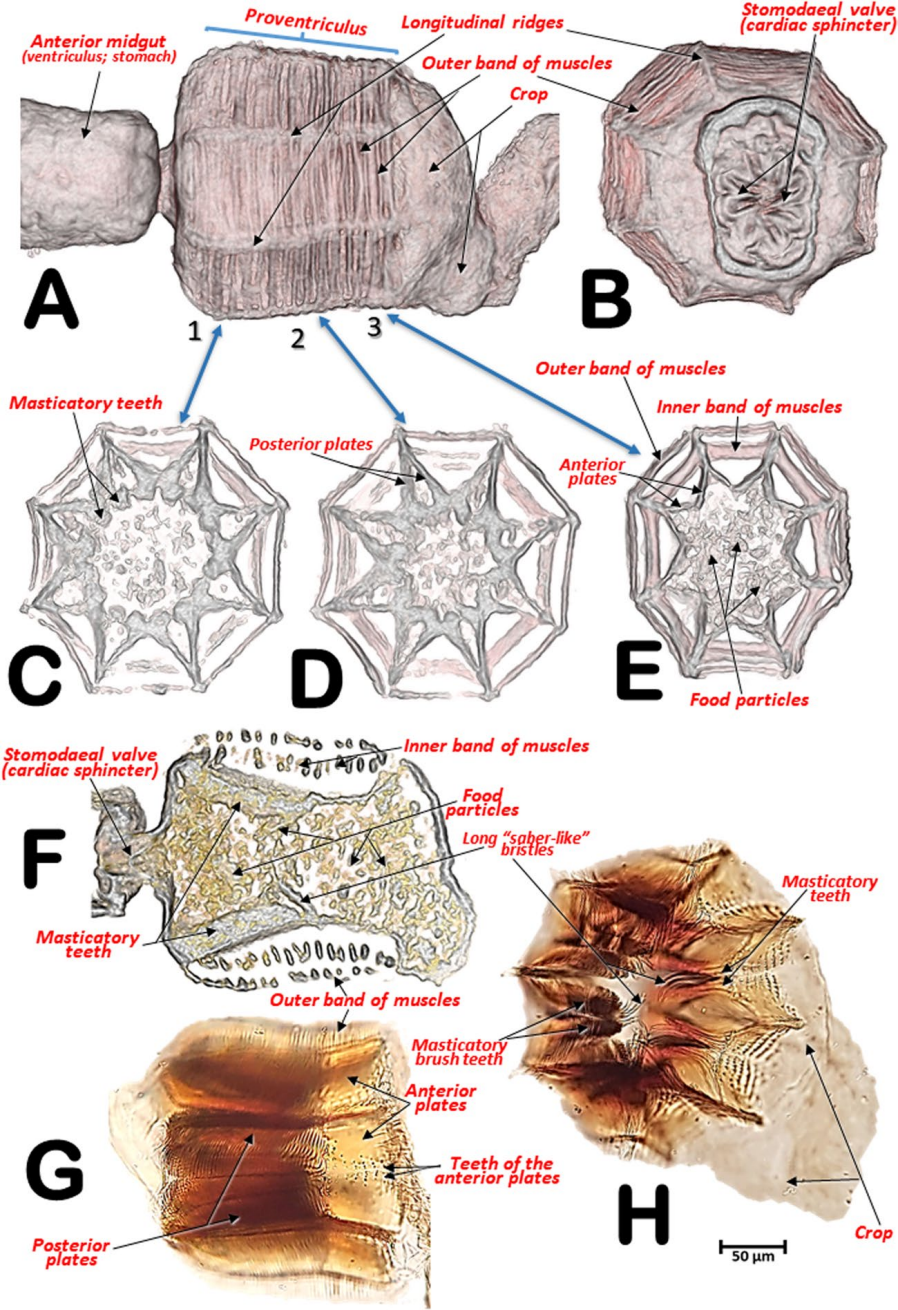
Details of the proventriculus: micro-CT volume-rendered images (**A–F**) and light microscopy photographs (**G**,**H**). Right-lateral view (**A**,**G**); posterior view showing the stomodaeal valve (=cardiac sphincter) (**B**). Progressive transverse slices (thickness ca. 20 µm) from the basal to the apical zone, each marked with arrows numbered 1 to 3 (**A**,**C**,**D**,**E**); sagittal mid-slice (**F**); postero-lateral view (**H**). Most of the nomenclature after Eaton^50^.

**Figure 7:**
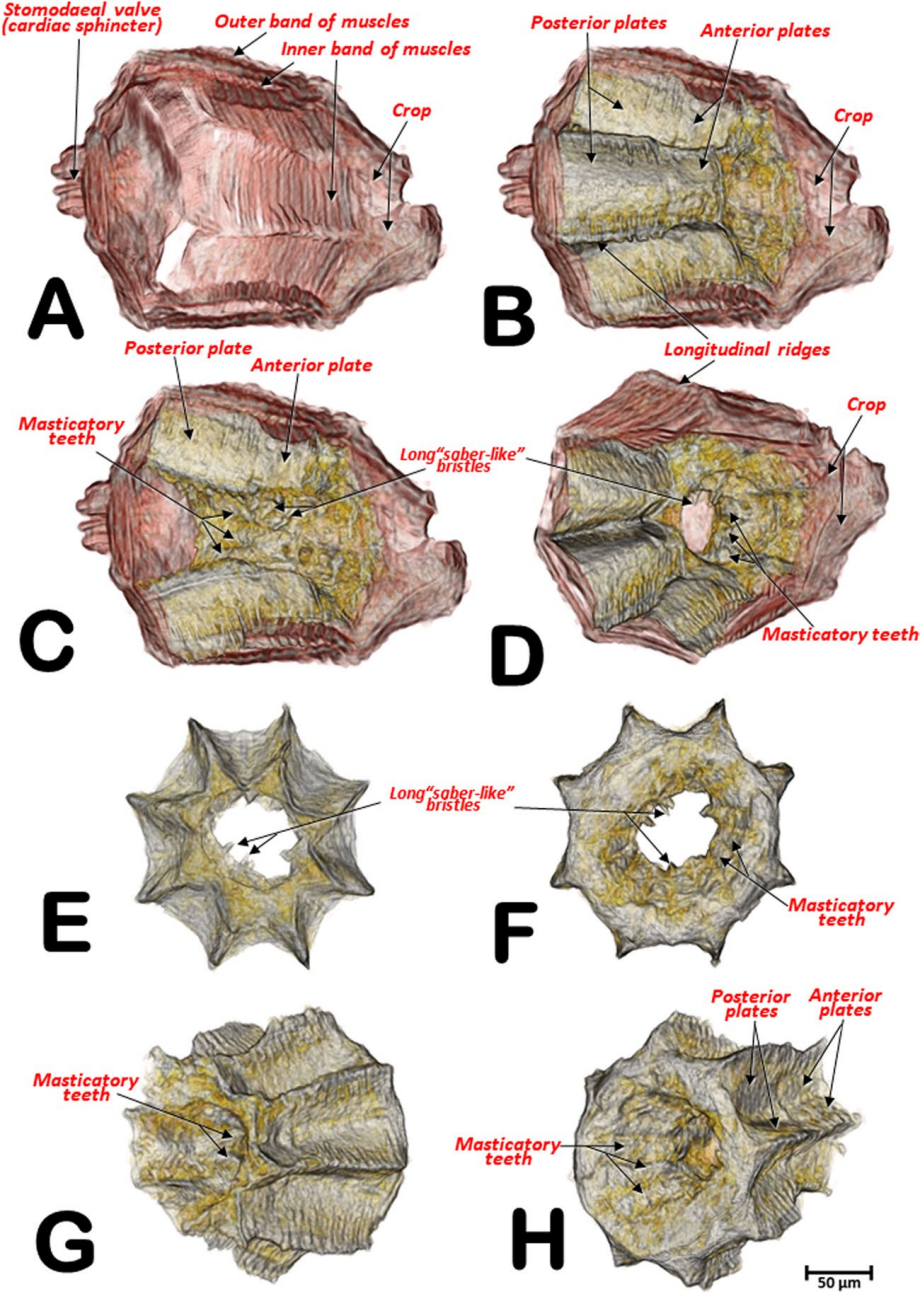
Micro-CT volume rendered images of the internal structures of the proventriculus from different perspective views: right-lateral (**A**,**B**,**C**), right-antero-posterior (**D**), anterior (**E**), posterior (**F**), left antero-posterior (**G**) and right-postero-anterior (**H**). Different perspectives of the grinding sclerotized complex isolated (**E–H**). **A**: a superficial window was opened, and the grinding sclerotized complex removed; **B**,**D**: as **A** but including the grinding complex; **C**: as **B** but the grinding complex has been superficially sagittally cut, eliminating one side of the octagonal structure.

The general arrangement of the internal structures and organs of both sexes can be seen in Figs. 2,3, and Supplementary Videos S1, S9. The digestive system, including the excretory system, are shown in Figs. 4, 5, and Supplementary Videos S1–S3, S9. Differences between the sexes in the shape of the midgut and the trajectories of the convolutions that make up the midgut and hindgut are shown in Fig. 4G,H, and Supplementary Video S2. Detailed views of the external and internal structures of the proventriculus, including the musculature are shown in Figs. 6, 7. Light microscopy views are included (Fig. 6G,H) for comparison with the micro-CT reconstructions (Fig. 6A–F; Supplementary Videos S3, S9).

**Figure 8:**
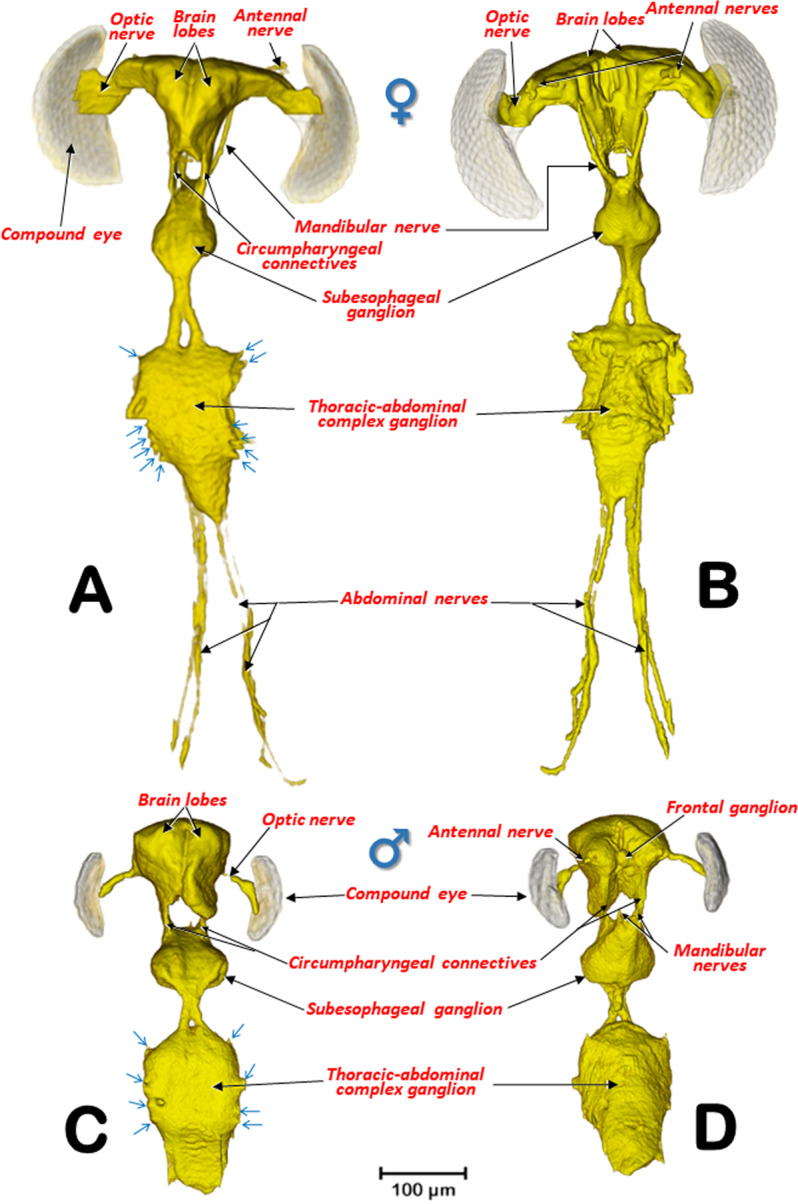
Nervous systems of a female (**A**,**B**) and a male (**C**,**D**) coffee berry borer, showing the main ganglia and nerves (**A**,**C**: dorsal; **B**,**D**: ventral). The abdominal nerves are not shown in the male. The nerve insertions in the thoracic-abdominal-complex ganglion are indicated with blue arrows. Terminology after Atkins and Chapman^75^ and Snodgrass^45^.

**Figure 9:**
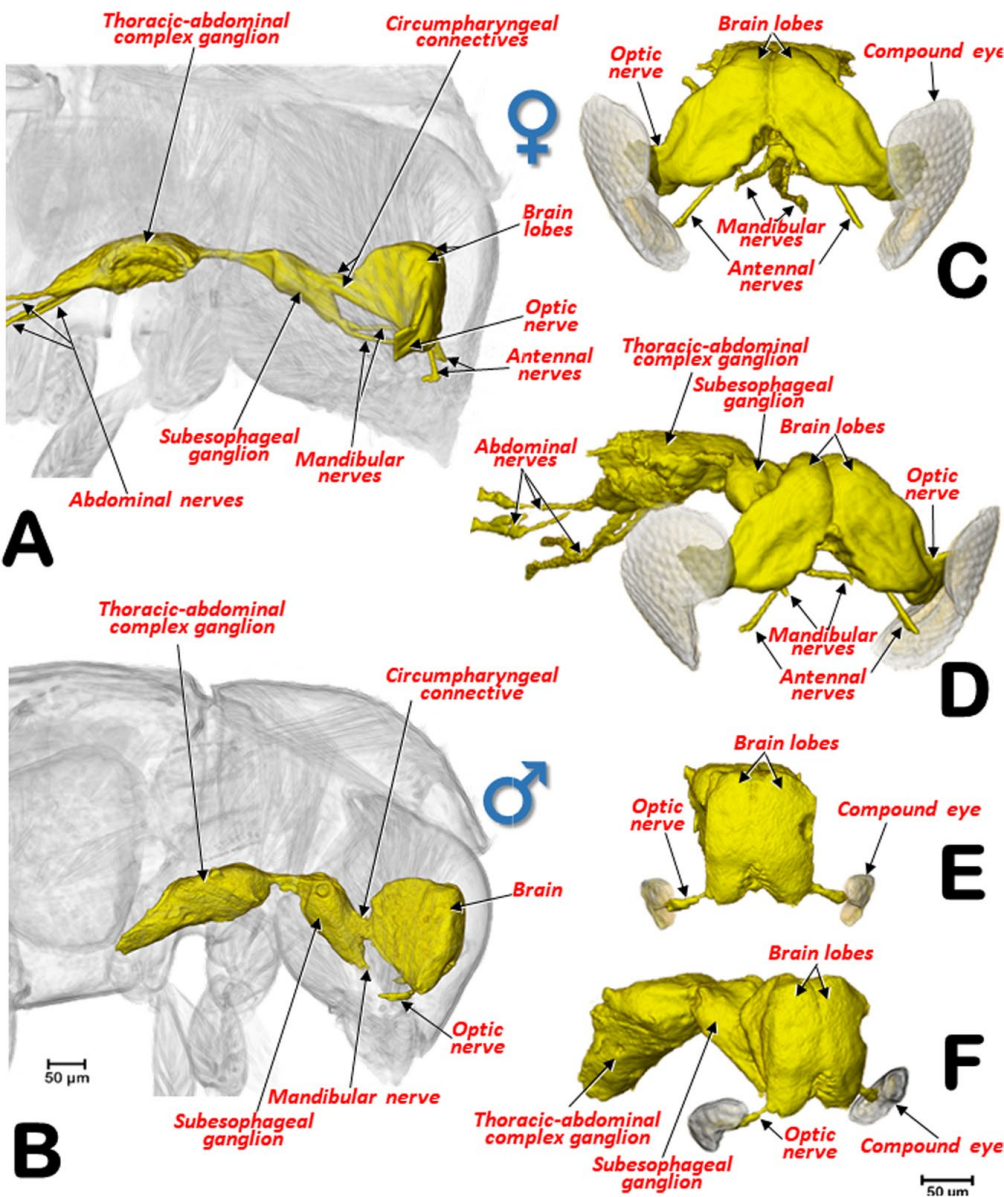
Lateral views of the nervous system of a female (**A**) and a male (**B**) coffee berry borer allowing comparison of the relative size of the central nervous system in relation to the head. Details in frontal (**C**,**E**) and latero-frontal (**D**,**F**) views of the central nervous system of a female (**C**,**D**) and a male (**E**,**F**) focusing on the differences between sexes.

**Figure 10:**
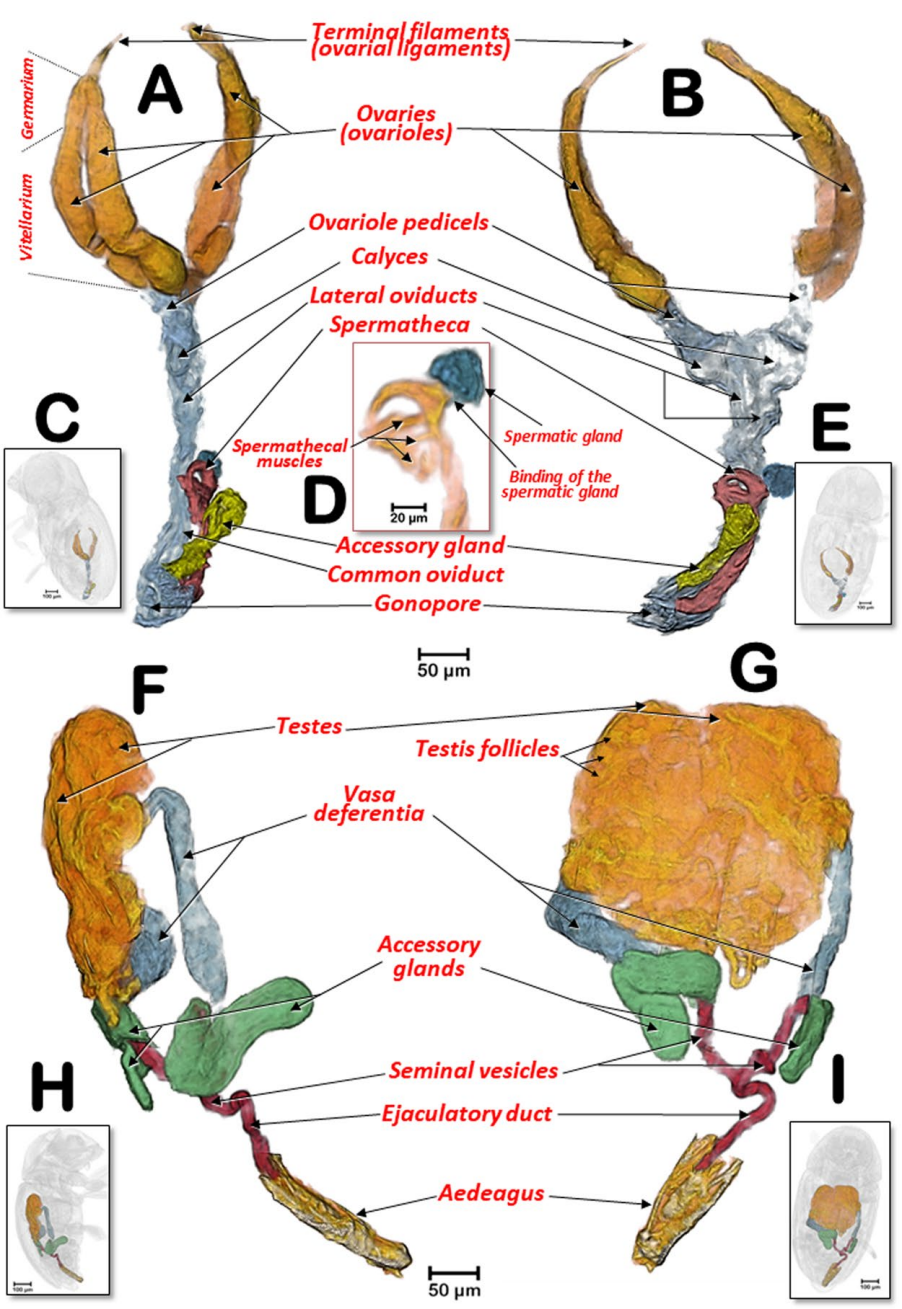
Female (**A–E**) and male (**F–I**) coffee berry borer reproductive systems, in latero-dorsal (**A**,**C**), lateral (**E**,**G**) and dorsal (**B**,**D**,**F**,**H**) views. Details of the distal part of the spermatheca, with the spermathecal muscles and the spermatic gland (**D**). Figures (**C**,**E**,**H** and **I)** show the positions of the insect corresponding to figures (**A**,**B**,**F** and **G**), respectively.

**Figure 11:**
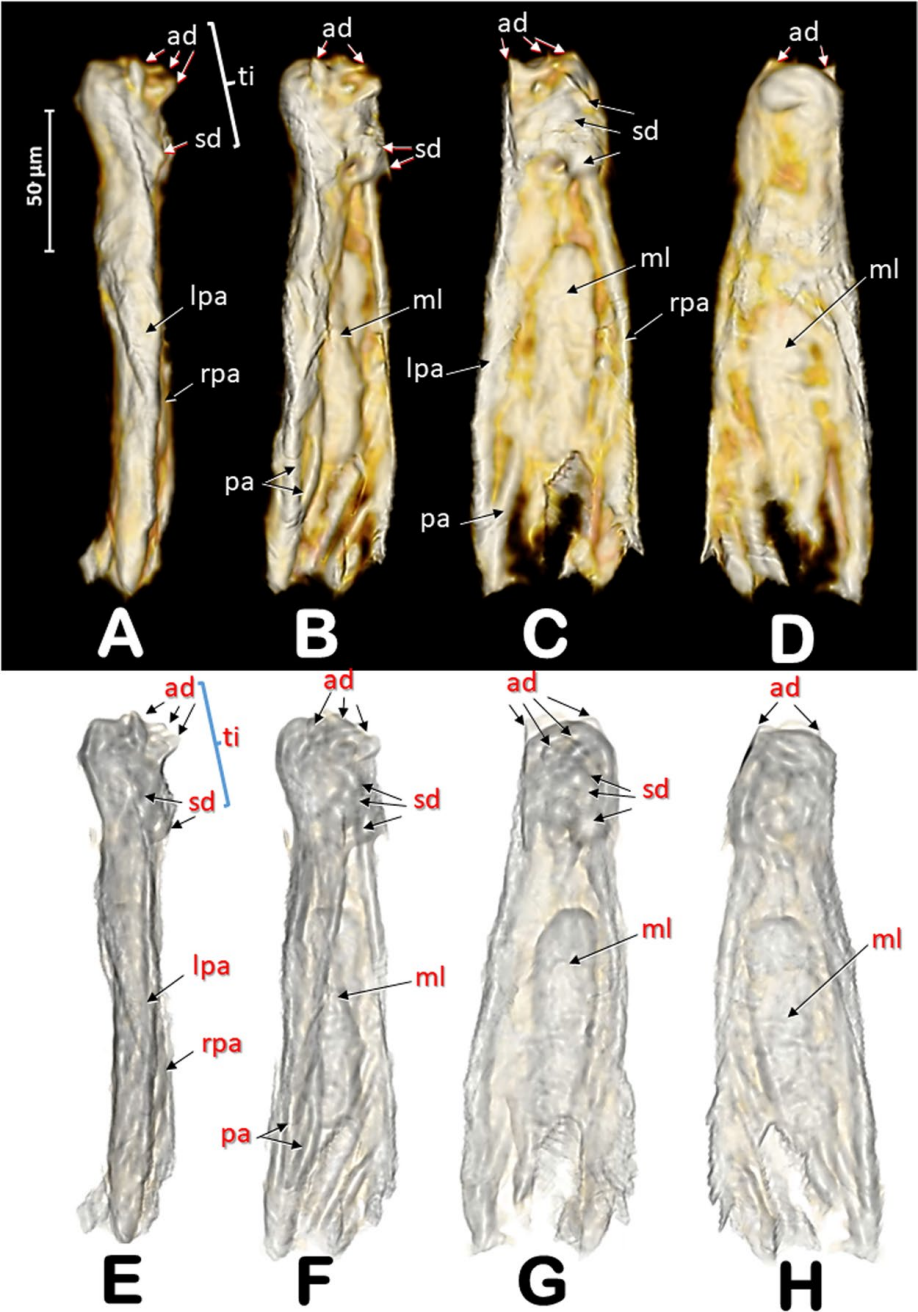
Rendered images of different views of the aedeagus: left-lateral (**A**,**E**); left-latero-dorsal (**B**,**F**); dorsal (**C**,**G**); and ventral (**D**,**H**). Images at the bottom have been rendered transparent by software to enhance the view of internal structures. Abbreviations: ad = apical denticles (titillators); lpa = left parameres; rpa = right parameres; ml = median lobe (‘penis’); pa = paramere apophysys; rpa = right paramere; sd = subapical dorsal denticles; ti = titillators. Terminology after Lindroth and Palmen^70^.

The general internal anatomy of an adult female (with organs and muscles) is shown in Fig. 5A, with the dorsal vessel, aorta, and the nervous system highlighted in different colours. The nervous system and its positional relationship with the digestive system is shown in Fig. 5, and Supplementary Videos S4, S9. Detailed views of the nervous system in both sexes are shown in Figs. 8, 9. The female and male reproductive systems are shown in Fig. 10 and Supplementary Video S5. A study of the aedeagus is shown in Fig. 11 and Supplementary Videos S6, S7.

## Discussion

The presence of eight tergites in the male has been described previously^7,8^ (Fig. 1A) but the presence of only seven in the female is new (Fig. 1B). Nüsslin^37^ stated that *Hypothenemus* (Cryphalini) females had eight tergites while Wood^38^ stated in reference to the 8^th^ tergite that “In all other scolytids and most curculionids it is of reduced size, lacks pubescence and is telescoped beneath and hidden by tergum 7”; this has been reported for various bark beetle species^38–41^. As part of this study, we dissected ten females reared in artificial diet and did not find a covered/obscured 8^th^ tergite. Therefore, it is possible that, either there is variation in the number of tergites amongst the 181 species of *Hypothenemus*^5^, or the 7^th^ and 8^th^ tergites are fused, as was reported in *Conophthorus* (Scolytini)^41^. Interestingly, there has been one report of a male coffee berry borer reared on artificial diet that had only seven tergites (Fig. 2C in Vega *et al*.^42^). However, in our study, we dissected ten males from the same colony and all of them had eight tergites.

A previously unrecorded external characteristic that varied between the sexes in our study occurred in lateral view, i.e., the outline of the pronotal disc in males was uniformly curved (Fig. 1B) while in females it had a slight concave depression (Fig. 1D). As has been previously described, coffee berry borer males are smaller than females, have vestigial wings^5,42^ and rudimentary compound eyes^43^ (Figs. 1, 8, 9E,F; Supplementary Videos S1, S4). Other characteristics that vary between sexes in bark and ambrosia beetles are discussed by Kirkendall *et al*.^44^ and, for *Hypothenemus* in particular, by Wood^38^ and Vega *et al*.^5^.

The alimentary canal, from mouth to anus, consisted of three main parts: foregut (stomodaeum), midgut (mesenteron) and hindgut (proctodeum). In contrast to the midgut, the foregut and the hindgut were ectodermic derivates and chitinized. The foregut was comprised of the preoral cavity, the mouthparts (where food is masticated), the pharynx, and the esophagus, which was enlarged posteriorly into the crop (where food is temporarily stored before entering the proventriculus, a gizzard-like organ located under the prothorax and whose main function is to grind and filter food particles). From there, food enters the anterior midgut (also referred to as stomach and ventriculus) and eventually the hindgut. Waste is excreted through the posterior opening of the alimentary canal (i.e., the anus). The insertion of Malpighian tubules was coincident with the limit between the mesodeum (midgut) and the proctodeum (hindgut) separation, as described by others^45–48^.

The pharynx had chitinized ridges, where the pharynx dilator muscles were inserted, and opened into the preoral cavity. The preoral cavity ran across the head, below the brain (Figs. 2, 3, 5; Supplementary Videos S1, S2), through the nervous system (Fig. 5; Supplementary Videos S4, S9), connecting with the crop via a short esophagus. The crop was comprised of two contiguous cavities, one small anterior one and another more voluminous one connected to the proventriculus (Figs. 2, 3, 4, 5, 6A; Supplementary Videos S2, S9).

The proventriculus (Fig. 6, Supplementary Videos S3, S9) was an octagon-shaped organ and once thought to have taxonomic value in Scolytoidea^48–51^. A series of outer band muscles surrounded eight heavily sclerotized units (Figs. 6, 7), as previously described by Eaton^50^ for *Ips mexicanus* (=*radiatae*) (Hopkins); these muscles exert a peristaltic function (contraction and relaxation) that grinds and moves food particles through the proventriculus. Another band of inner muscles also have a peristaltic function on the grinding structures, formed by eight plates that appeared as a whole and in cross section with a starry arrangement (Figs. 6C–E, 7E,F). Each of these were formed in two parts, an anterior plate and a posterior plate. Internally these plates formed masticatory teeth, which triturated food particles. The presence of long ‘saber-like bristles’ and masticatory brush-teeth also assist in food grinding and filtering (Figs. 6C–H, 7; Supplementary Videos S2, S3). The general structure agreed with Calders’s^52^ ‘Type 7’ Scolytinae.

Eaton^50^ described the grinding-straining mechanism of food moving through the proventriculus of *I. mexicanus* as follows: “Its passage through the proventriculus is probably restricted to the side channels formed by the rows of lamellae, since the obstructing brushes extending into the lumen would prevent direct movement of the food material through the central opening” and indicated this probable passage of food in Fig. 8 (p. 46). However, in our study of the coffee berry borer, it could be clearly seen that the lumen was filled with food particles while the spaces marked by Eaton^50^ as possible passageways for food, were devoid of food particles (Fig. 6C–F).

From the proventriculus the food moves through the cardiac sphincter (also known as the cardiac or stomodaeal valve; Fig. 6B–F) to the anterior midgut. There were differences between the sexes in the anterior midgut. In the male coffee berry borer, the anterior midgut was much dilated anteriorly (Figs. 2B, 4A–C,G; Supplementary Video S2), which was not apparent in the female (Figs. 3B, 4D–F,H; Supplementary Videos S2, S9). After scanning eight females and two males, it was clear that males had a conspicuously dilated midgut that was not present in females and this was not dependent on how full the cavity was with food. Moreover, we observed that the female (but not in the male) had short papillae covering the entire surface of the anterior part of the midgut, close to the stomodaeal valve; these were also present on 2/3 of the ventral surface (Fig. 4I,J). This arrangement of papillae has not been described previously in curculionids^52^.

The midgut has no chitinous lining and is the primary site for secretion of digestive enzymes into the lumen and for absorption of nutrients^53^, although it should be noted that salivary enzymes (e.g., amylases) secreted in the foregut can also initiate digestive action^54^. There is considerable variation in the pH of the foregut, midgut and hindgut of Coleopteran species^53^, with the midgut and hindgut of the coffee berry borer having an acidic pH (4.5–5.2)^55^. Aggregation pheromones in *Dendroctonus* and *Ips* are also produced in the midgut^56^.

The transit of food through the alimentary canal in the coffee berry borer takes ca. 24h^57^. The anterior midgut of the coffee berry borer has a gradient in oxygen content, with a microaerophilic region close to the surface (0–300 µm depth) followed by an anaerobic region (400–600 µm depth)^57^. This gradient allows for the activity of oxygen-dependent and anaerobic enzymes. For example, N-demethylases (involved in caffeine metabolism in the coffee berry borer) are oxygen-dependent^58^, while the fermentation of polysaccharides such as mannan in the coffee bean^5,59,60^, is an anaerobic process^61^.

The posterior midgut had two diverticula (right and left), also known as gastric caeca (Figs. 2–4, 5B; Supplementary Videos S2, S9); these increase the surface area of the midgut and are involved in water absorption, digestive activity, and uptake of nutrients^54^.

Insertion of the Malpighian tubules is recognized as the beginning of the hindgut^45–47,62^. Insertion of six Malpighian tubules has been reported in other Scolytinae^51,63–67^ with the distal portion of the Malpighian tubules embedded in the tissues surrounding the rectum^65–67^. However, after exhaustive visualization of these tubules in both sexes of the coffee berry borer, we only observed four long Malpighian tubules, in contrast with the six reported previously^11^. They appeared to be folded several times, going forwards and backwards (Figs. 2, 3, 4A–F, 5C,D; Supplementary Videos S1, S2, S4, S9); the distal portions, which were situated at the level of the anterior midgut (stomach), did not surround the rectum. The distal portions are immersed in haemolymph and serve a urinary function (i.e., as excretory organs that remove nitrogenous wastes, mainly uric acid) and also balance water and salt levels in the haemolymph. The anterior part of the hindgut is also referred to as the ileum or intestinum, and the posterior part in some insects is divided into colon and rectum (these three parts are also known as pylorus, ileum and rectum^47^, and are responsible for absorption of solutes such as amino acids, salts and water^46,47,68^. The rectum is the most dorsal part of the digestive system, and was slightly displaced to the right side, running backwards to open externally at the anus (Figs. 2–4, 5B–D; Supplementary Videos S1, S2, S4, S9).

It has been reported that the length of the alimentary canal in *Dendroctonus armandi* is almost three times the length of the insect^67^. In the coffee berry borer, the length of the alimentary canal was 2.82 mm for the male and 3.48 mm for the female; the ratio of the length of the alimentary canal to the body of the insect was 2.9 and 2.3, for the male and female, respectively. Moreover, we observed additional differences between the sexes in the number of trajectories of the midgut and hindgut (based on the number of times the alimentary canal began a new trajectory curve). We observed nine different trajectories in the male compared with eight in the female (Fig. 4G,H; Supplementary Video S2). Males have a shorter body length than females, suggesting that additional folding of the tubes of mid- and hindgut was necessary to fit into the smaller space. In both sexes a rectal ampulla was connected to the final tract of the rectum (Figs. 2–3, 4A–F; Supplementary Videos S1, S2); in other Coleoptera this ampulla has a defensive function via secretions^53^.

The male reproductive system (Figs. 2, 10F–I, 11; Supplementary Videos S1, S5) was comprised of two testes touching medially and positioned above the midgut (containing lobular testis follicles where spermatozoa are formed^46,47^). Each testis had a vas deferens and a single elongated accessory gland, in contrast to three pairs of accessory glands per pair of testes in *D. monticolae*^69^. Accessory glands are involved in the production of seminal fluids that mix with sperm to form the ejaculate;^45^ the vas deferens dilates to form a small seminal vesicle for storage prior to ejaculation^47^. The vasa deferentia on both sides meet to form an ejaculatory duct that is connected to the aedeagus.

The structure of the aedeagus in insects was studied in-depth by Snodgrass^45^ and updated for Coleoptera by Lindroth and Palmen^70^. The aedeagus is an important taxonomical characteristic used for species identification of insects in general and is of particular relevance in studies of Coleoptera^71^ including bark beetles^72^. However, to the best of our knowledge, there has been no previous detailed description of the aedeagus in the coffee berry borer. There are only draft schematic drawings and not detailed photographs^10,11^. The micro-CT rendered images of the aedeagus of the coffee berry borer (Figs. 2A,B, 10F–I, 11; Supplementary Videos S6, S7) show two lateral lobes (parameres) and a medial lobe (the intromitting structure, the penis) between them, with a rounded distal part opening called the gonopore. The distal extremity of the aedeagus is armed with apical spines and subapical-dorsal denticles that, as a whole, form anchoring structures (known as titillators^45^) helping ensure the sexes remain connected during copulation. The general structure of the aedeagus corresponds to the vaginate type described by Lindroth and Palmen^70^ where the parameres are elongated, forming a dorsal channel through which the penis moves.

The female reproductive system (Figs. 3, 10A–E; Supplementary Videos S1, S5) is comprised of two ovaries (located at each side of the body, slightly beneath the hindgut), each containing two ovarioles. Each ovariole had a terminal filament forming the suspensory apparatus of the mature ovary;^45^ a germarium in which oocytes are produced from oogonia; a vitellarium in which yolk is deposited into the oocytes through a membranous tube;^47^ and the ovariole pedicel that enlarges to form the calyx for the reception of eggs^45^. From each calyx emerged a short lateral oviduct; both lateral oviducts joined to form the common oviduct leading to the female gonopore. Close to the end of the common oviduct, on the right side, in a dorsal position was an ampulla-shaped accessory gland; between the accessory glands (very close to each other) was a hook-shaped spermatheca. The micro-CT rendered images obtained here were totally coincident with light microscopic images obtained following dissection^10,11,14^. Details of the hooked distal part of the spermatheca, spermatic gland, and the spermathecal muscles described recently^14^ were enhanced using micro-CT (Fig. 10D; Supplementary Video S8). Román-Ruiz *et al*.^14^ first photographed and described the spermatic gland as a “cell agglomeration on the curved distal end of the spermatheca”, but they added that “a further study will be necessary to confirm this finding”. The micro-CT reconstructions allowed us to visualize the structure previously identified as the spermatic gland (Fig. 10A,B,D; Supplementary Video S8) thus confirming its existence and structure.

The circulatory system in insects is responsible for the movement of haemolymph into the haemocoel spaces where organs are immersed. The haemolymph enters various contractile chambers of a dorsal vessel (heart) and is pumped forwards through an anterior aorta. This is what it is considered as an open circulatory system. The circulatory system does not have a key role in the transport of gases as this is achieved by an aero-vascular system formed by a complex of tubes (tracheal system)^45–47,73^. In the coffee berry borer, as in other insects, the circulatory system is quite simple in contrast with the complexity of the tracheal respiratory system^19^. The dorsal vessel was segmented in the female; it was in a medial position within the abdomen, just below the tergites. The dorsal vessel had three conspicuous dilated chambers beneath tergites 4–6 which were prolonged anteriorly and posteriorly by aortic vessels.

When observing the musculature (Figs. 1E,F, 2, 3, 5A), it is noteworthy that the dorso-longitudinal and dorso-ventral flight muscles were clearly visible in the female (Figs. 1F, 3A, 5A) and absent in the male (Figs. 1E, 2A). This is likely to be because males have lost their ability to fly as they have vestigial wings, spending their entire life inside the coffee berry^5,42^. The micro-CT rendered images of the flight muscles are completely comparable to, and actually clearer, than those previously published from dissected insects^13^. Various muscles connected to the pharynx and mouthparts were clearly visible inside the head (mandibular, pharyngeal, maxillary and labial muscles; Figs. 2A, 5A). Head flexor and extensor muscles (Fig. 5A) were clearly visible in the pronotum. While we do not describe here every muscle visible in the micro-CT rendered images and supplementary videos, it will be the subject of a future paper.

The anatomy of the nervous system of insects was described extensively by Snodgrass^45^ and summarized and updated by Grassé^74^. There have been several studies on the anatomy of the nervous system in different families and species of Coleoptera^53,74^. To our knowledge, despite the extensive revision by Calder^52^, the most detailed anatomical studies of the nervous system of bark beetles is that of *D. pseudotsugae* by Atkins and Chapman^75^, and our own study of the central nervous system of the coffee berry borer (Figs. 2A, 3A, 5,8,9; Supplementary Videos S1, S4, S9); both showed similar general patterns of organisation.

In the coffee berry borer, the brain (Figs. 2A, 3A, 5), located above the anterior end of the foregut, was connected to a ventral cord by two circumpharyngeal connectives (Figs. 5, 8, 9A,B). Most of the ganglions in the ventral cord were fused, so it was only possible to distinguish a subesophageal ganglion (located beneath the esophagus in a ventral posterior cephalic position; Figs. 5, 8, 9A,B,D,F) and a large thoracic-abdominal complex ganglion (in a ventral thoracic position just below the proventriculus; Figs. 2A, 3A, 5, 8, 9A,B,D–F). Other Scolytinae species have a prothoracic separated ganglion^52,75^. In other species, the connectives linking the brain to the ventral cord are known as circumesophageal connectives^45–47,75^. However, it is clear that, in the coffee berry borer, these connectives surround the pharynx and not the esophagus (Figs. 2A, 3A, 5; Supplementary Videos S4, S9). For this reason, we describe them as circumpharyngeal connectives.

Micro-CT also allowed us to visualize different pairs of nerves including optic (to innervate eyes) and antennal (to innervate antennae) nerves in the brain; and mandibular nerves originating in the subesophageal ganglion (to innervate the mandibles) (Figs. 5A, 8A,B,D,9A,B,D). Nerve insertions into the thoracic-abdominal-complex ganglion and long abdominal nerves were also clearly visible (Figs. 8A–C, 9E); together, these innervate the wings, legs and abdominal muscles. Note that the abdominal nerves in the male were not segmented and have not been included in figures. Details of the different ganglia, such as the frontal ganglion (situated ventral to the brain) (Fig. 8D) were also visualized (Figs. 8, 9).

Sexual differences in the nervous system of insects has been reported, both in morphology, size and in the structure of the neural connections, e.g., in social insects^76^ and flies^77^. In the coffee berry borer, there were conspicuous differences both in shape, size and relationship to the head of the nervous system, between the female and the male. The female had a larger, more laterally extended brain lobes, and thicker optic lobes (Fig. 9C,D) than the male (Fig. 9E,F). In relation to the size of the head, the brain of the female is smaller (Figs. 3A, 9A) than the male brain (Figs. 2A, 9B). The smaller brain and thinner optic nerves observed in the male is related to the biology of the species; males remain inside the coffee berry and have rudimentary compound eyes, vestigial wings, and flight muscles, as discussed above.

## Conclusions

The use of micro-CT to elucidate the anatomical structures and organs of the coffee berry borer has facilitated a complete reconstruction of the anatomy of the insect, revealing the actual position of internal structures and organs without the distortions encountered using dissection methods. The technique has also allowed us to obtain detailed rendered images and videos of the aedeagus, which we used to describe it for the first time. This study is the first complete micro-CT reconstruction of the anatomy of an insect and is also the smallest insect to have been evaluated in this way. Moreover, we report previously unreported differences between the sexes with respect to both external morphology (lateral outline of the pronotum and number of abdominal tergites), and internal anatomy (flight musculature, midgut shape, hindgut convolutions and the shape and size of the brain). We have included supplementary videos and a 3D model that is suitable for use with mobile devices and could be a useful tool for scientists working on other insects as well as a teaching aid.

## Methods

### Insects

Coffee berry borers were reared in artificial diet at the USDA, ARS, Beltsville laboratory^78^.

### Micro-CT scans

The specimens were killed by submerging them in 30% ethanol for 25 minutes. They were then dehydrated in an ethanol series (50%, 70%, 80%, 90%; 25 minutes in each concentration), and stained in 1% iodine in absolute ethanol for three days. Stained insects were placed in a well and submerged in hexamethyldisilazane for 4 h and air-dried overnight. For the scans, specimens were glued with cyanoacrylate to the tip of a nylon fishing line 200 μm in diameter as described by Alba-Tercedor^79^ and scanned using a Bruker SkyScan 1172 microtomograph (Bruker-micro CT, Kontich, Belgium) with a Hamamatsu L702 X-ray source and a Ximea 11 megapixels camera. The setting parameters were as follows: voltage = 49 kV; current = 64 µA; isotropic voxel size = 0.52 µm; image rotation step = 0.5° for the male and 0.4° for the female; 360° of rotation scan, and no filter, resulting in a scan duration of 2 h: 47 min: 55 s and 722 × -ray images for the male, and 3 h: 29 min: 39 s and 902 × -ray images for the female.

### Image reconstruction, measurements and supplementary videos

Bruker micro-CT’s Skyscan software (NRecon, DataViewer, CTAnalyser) was used for primary reconstructions and the ‘cleaning’ process to obtain datasets for ‘slices’ as described previously by Alba-Tercedor^79^. Volume-rendered images and Supplementary Videos S1–S8 were obtained using Amira’s software, v. 6.7.0 (Thermo Fisher Scientific, Waltham, MA)^80,81^. The built-in ‘volrenRed.col’ colour filter was selected to obtain volume-rendered reconstructions in Figs. 1A,C, 6A–F, 7, 10. Different anatomical parts were independently segmented to finally obtain the rendered colorized images of Figs. 2–10. To be able to obtain the actual texture of structures in desired colours, after segmentation, each structure was subjected to the following arithmetic operation: A*(B > *0)*, where *A* represents the whole animal and *B* the segmented structure. The total length of the alimentary canal was measured manually from the oral opening to the anus using Amira’s measuring tool. Figs. 4I,J, Supplementary Video S9 and Supplementary 3D models S10 and S11 for both sexes were obtained using CTVox (a Bruker micro-CT’s Skyscan software). Colours were obtained by adjusting the transfer function curves in accordance with the transparency of the structures to X-ray, as described by Alba-Tercedor^79^.

### Light microscopy study of the proventriculus

A female was cleared by submersion in 10% aqueous KOH for 48 h at room temperature, followed by dissection and subsequent mounting on a slide in a variation of Hoyer’s liquid^82^. Figure 6G,H were obtained using a Samsung Note 8 smartphone connected to the ocular of an Olympus CH-2 binocular microscope.

## Supplementary Information

Supporting Information

S1

S2

S3

S4

S5

S6

S7

S8

S9

S10

S11

## Data Availability

The datasets generated and analyzed during the course of the study are available from J.A.T. upon reasonable request.
