# Publisher Correction: Anatomical study of the coffee berry borer (*Hypothenemus hampei*) using micro-computed tomography

**DOI:** 10.1038/s41598-019-56813-0

**Published:** 2019-12-24

**Authors:** Ignacio Alba-Alejandre, Javier Alba-Tercedor, Fernando E. Vega

**Affiliations:** 1 Department of Zoology, Faculty of Sciences, University of Granada, Campus de Fuentenueva, 18071 Granada, Spain; 2 Sustainable Perennial Crops Laboratory, United States Department of Agriculture, Agricultural Research Service, Beltsville, MD 20705 USA

Correction to: *Scientific Reports* 10.1038/s41598-019-53537-z, published online 20 November 2019

In the original version of this Article, nine Supplementary Video files were omitted. This has been corrected in the HTML version of the Article; the PDF version was correct at the time of publication.

