# Supplementary material for: Anatomical study of the coffee berry borer (Hypothenemus hampei) using micro-computed tomography
Source: Sci Rep. 2019 Nov 20;9:17150. doi: 10.1038/s41598-019-53537-z (PMC6868283; doi:10.1038/s41598-019-53537-z)
Supplement: Supplementary file 1 — Supporting Information [file 41598_2019_53537_MOESM1_ESM.pdf]

Supplementary Information

Anatomical study of the coffee berry borer (*Hypothenemus hampei*) using micro-computed tomography

Ignacio Alba-Alejandre[1], Javier Alba-Tercedor[1,*] and Fernando E. Vega[2,*]

[1]Department of Zoology, Faculty of Sciences, University of Granada, Campus de Fuentenueva, 18071-Granada, Spain

[2]Sustainable Perennial Crops Laboratory, United States Department of Agriculture, Agricultural Research Service, Beltsville, MD, 20705, USA

*Correspondence and requests for materials should be addressed to J.A.T. (email: jalba@ugr.es) or F.E.V. (email: Fernando.Vega@usda.gov)

Supplementary Videos (mp4):

S1. Animated volume-rendered images of both sexes of the coffee berry borer (*Hypothenemus hampei*) showing the general anatomy.

S2. Comparative animated volume-rendered images of the digestive system in both sexes of the coffee berry borer.

S3. Animated volume-rendered images of the detailed anatomy of the proventriculus of the coffee berry borer showing the circulatory, digestive and nervous system.

S4. Animated volume rendered images of a female coffee berry borer showing the circulatory, digestive and nervous system.

S5. Comparative animated volume-rendered images of the reproductive system in both sexes of the coffee berry borer.

S6. Animated volume-rendered images of the aedeagus of the coffee berry borer.

S7. 3D animated volume-rendered images of the aedeagus of the coffee berry borer. To visualise the 3D effect, please use red-cyan anaglyph glasses.

S8. Animated volume-rendered images of the female spermatheca and spermatic gland of the coffee berry borer.

S9. Animated volume-rendered images of the internal organs of a female coffee berry borer. With detailed views of different structures, enhancing the digestive system

(proventriculus, stomomodaeal valve, intestinum, gastric caeca and internal papillae of the midgut) and its location in relation to the nervous system.

Supplementary 3D model for use with mobile devices (vxm):

S10. Adult male coffee berry borer.

S11. Adult female coffee berry borer.

These can be visualized using the CTvox app for mobile devices (smartphones and tablets, either with iOS or Android systems). To install CTvox on your device, go through Apple’s App Store/Google Play Store, in the usual fashion (the app is free of charge). Instructions can be downloaded at the following links:

1.- For iOS devices:

a) For Ipad:

https://www.bruker.com/fileadmin/user_upload/8-PDF-Docs/Microtomography/CTvoxForIpad.pdf

b) For Iphone:

https://www.bruker.com/fileadmin/user_upload/8-PDF-Docs/Microtomography/CTvoxForIPhone.pdf

2.- For Android devices:

https://www.bruker.com/fileadmin/user_upload/8-PDF-Docs/Microtomography/CTvoxForAndroid.pdf
